# Enzymatic Degradation of PrP^Sc^ by a Protease Secreted from *Aeropyrum pernix* K1

**DOI:** 10.1371/journal.pone.0039548

**Published:** 2012-06-28

**Authors:** Marko Šnajder, Tanja Vilfan, Maja Černilec, Ruth Rupreht, Mara Popović, Polona Juntes, Vladka Čurin Šerbec, Nataša Poklar Ulrih

**Affiliations:** 1 Biotechnical Faculty, University of Ljubljana, Ljubljana, Slovenia; 2 Blood Transfusion Center of Slovenia, Ljubljana, Slovenia; 3 Medical Faculty, University of Ljubljana, Ljubljana, Slovenia; 4 Veterinary Faculty, University of Ljubljana, Ljubljana, Slovenia; 5 Centre of Excellence for Integrated Approaches in Chemistry and Biology of Proteins (CipKeBiP), Ljubljana, Slovenia; Ohio State University, United States of America

## Abstract

**Background:**

An R30 fraction from the growth medium of *Aeropyrum pernix* was analyzed for the protease that can digest the pathological prion protein isoform (PrP^Sc^) from different species (human, bovine, deer and mouse).

**Methodology/Principal Findings:**

Degradation of the PrP^Sc^ isoform by the R30 fraction and the purified protease was evaluated using the 6H4 anti-PrP monoclonal antibody. Fragments from the N-terminal and C-terminal of PrP^Sc^ were also monitored by Western blotting using the EB8 anti-PrP monoclonal antibody, and by dot blotting using the C7/5 anti-PrP monoclonal antibody, respectively. For detection of smaller peptides from incomplete digestion of PrP^Sc^, the EB8 monoclonal antibody was used after precipitation with sodium phosphotungstate. Characterization of the purified active protease from the R30 fraction was achieved, through purification by fast protein liquid chromatography, and identification by tandem mass spectrometry the serine metalloprotease pernisine. SDS-PAGE and zymography show the purified pernisine plus its proregion with a molecular weight of *ca*. 45 kDa, and the mature purified pernisine as *ca*. 23 kDa. The purified pernisine was active between 58°C and 99°C, and between pH 3.5 and 8.0. The temperature and pH optima of the enzymatic activity of the purified pernisine in the presence of 1 mM CaCl_2_ were 105°C ±0.5°C and pH 6.5±0.2, respectively.

**Conclusions/Significance:**

Our study has identified and characterized pernisine as a thermostable serine metalloprotease that is secreted from *A. pernix* and that can digest the pathological prion protein PrP^Sc^.

## Introduction

The term ‘prion’ was first introduced by Prusiner in 1982. He defined it as a small proteinaceous infectious particle that can resist inactivation by nucleic-acid-modifying procedures. At first, the research was oriented towards identification of the agent causing scrapie in sheep and goats. Scrapie is a type of transmissible spongiform encephalopathy (TSE) that belongs to a group of diseases that have also been recognized in several other animal species, as well as in humans: the prion diseases.

It is believed that TSEs develop after the cellular prion protein (PrP^C^) undergoes structural changes. PrP^C^ is a monomeric, glycosylated protein that is attached to cell membranes through a glycosylphosphatidylinositol anchor [1], and it is highly conserved among mammals [2]–[5]. It is expressed in different cell types, with the highest expression levels in the brain of animals and human. However, its function has not yet been clearly established [6]–[16]. The key step in the development of TSEs is the accumulation of the pathological PrP isoform (PrP^Sc^) with a ß-sheet rich region, unlike the α-helices that are the predominant secondary structure of PrP^C^ [17], [18].

As a consequence of its conformational characteristics, PrP^Sc^ has some unique features, among which there is resistance to protease digestion, and to detergents, heat, UV and ionization radiation treatments [19]. The PrP^Sc^ isoform thus has an unusual resistance to conventional chemical and physical decontamination methods, which raises substantial medical and food-industry considerations [20]–[22]. For this reason, several reports on proteolytic decontamination of TSE agents have been published in recent years.

The majority of proteases that have been studied require additional chemical or physical treatments of brain homogenates to enhance their ability to digest this PrP^Sc^ isoform. Pretreatment of brain homogenates with 0.1 M NaOH or 2% sodium dodecyl sulfate (SDS) increases PrP^Sc^ susceptibility towards some commercially available proteases [23], [24]. Furthermore, only after PrP^Sc^ heat treatment to 115°C can *Bacillus licheniformis* PWD-1 keratinase digest PrP^Sc^ in homogenates of bovine spongiform encephalopathy (BSE) and scrapie-infected brain [25]. Similarly, after long incubation times, the thermally denatured amyloid recombinant ovine PrP^Sc^ isoform was only partially degraded when incubated with extracellular proteases from anaerobic thermophilic prokaryotes and from *Streptomyces* subspecies [26]. Several other microbial proteases have been tested for activity against PrP^Sc^ [27]–[29]. Recently, some lichen extracts containing unknown serine proteases have been shown to promote PrP^Sc^ degradation [30] and some earthworm proteases in water extracts can successfully degradate PrP^C^ [31].

In the present study, we show that a protein fraction prepared from growth medium (the R30 fraction) in which the hyperthermophilic marine archaeon *Aeropyrum pernix* has been cultivated has proteolytic activity against the PrP^Sc^ isoform of different species. In Western blotting and dot blotting, several PrP^Sc^ fragments were revealed using monoclonal antibodies against different PrP epitopes. This proteolytic activity is additionally demonstrated by intracerebral bioassays. Further analysis of this R30 fraction shows that this proteolytic activity is associated with the serine metalloprotease pernisine.

## Materials and Methods

### Strain and Growth Conditions

*A. pernix* strain K1 (JCM 9820) was used in this study. The cells were grown under aerobic conditions at 92°C in a medium containing yeast extract (1.0 g/L), peptone (5.0 g/L), Na_2_S_2_O_3_.5H_2_O (1.0 g/L), AZOO reef salt (34 g/L) and HEPES (20 mM), pH 7.0, as described previously [32].

### Preparation and Analysis of the R30 Extracellular Extract with Proteolytic Activity

A cultivation batch (6.4 L) of *A. pernix* was stopped after 40 h of growth, and the cells were removed by centrifugation at 10,000×*g* for 15 min; the supernatant (growth medium) was then filtered through 45 µm and 20 µm cellulose nitrate filters. This growth medium was concentrated *ca*. 1500-fold using a concentrator (Pall Corp.) with a 10-kDa molecular-weight cut-off, and ultrafiltered using YM10 membranes (Millipore). This concentrated growth medium was loaded onto a Superdex 200 preparative grade gel filtration column (GE Healthcare), equilibrated with phosphate-buffered saline (PBS), pH 8.0. Fast protein liquid chromatography (FPLC) was carried out on an Äkta Explorer system (GE Healthcare). The fractions with the highest proteolytical activities were collected and ultrafiltered using Amicon Ultra-4 filters (Millipore). All of the purification procedures were performed at 4°C. For the analysis of the protein content of this R30 fraction, it was subjected to SDS polyacrylamide gel electrophoresis (SDS-PAGE), according to Laemmli *et al*. [33]. After separation on 12% SDS polyacrylamide gels, the protein bands were stained with Simplyblue dye (Invitrogen), according to the standard manufacturer protocol. The protein molecular weights were calculated from the SDS-PAGE using the BioNumerics program (Applied Maths NV, Belgium). Selected protein bands were analyzed by Orbitrap–tandem mass spectrometry (MS/MS) (at the Jozef Stefan Institute, Slovenia).

### Immunodetection of PrP Fragments after Treatment with the R30 Fraction and Purified Pernisine

The proteolytic activity of the R30 fraction against PrP^Sc^ was tested on the post-nuclear fractions from several uninfected and infected brain homogenates. These post-nuclear fractions were prepared by homogenization of the brain tissue (10%; w/v) in PBS, with added 0.5% NP-40 and 0.5% sodium deoxycholate, using a HT1000 Potter homogenizer, with aliquots stored at –80°C. Prior to use, the brain homogenates were cleared of particulate matter by centrifugation at 5,000×*g* for 5 min, followed by centrifugation for 10 min at 15,000×*g* to remove the nuclear fraction.

The reaction mixture (final volume, 15 µL) contained the R30 fraction (0.2 U; see below), 3 µL post-nuclear fraction, and the appropriate amount of PBS. These mixtures were incubated at 92°C for the times specified. A reference reaction mixture that contained 0.75 µg proteinase K instead of the R30 fraction was incubated in parallel at 37°C.

The reactions were stopped by the addition of 2× Laemmli sample buffer, and 20 µL was used for standard 12% SDS-PAGE. The gels were run at 120 V for 55 min, and then the proteins were transferred to nitrocellulose membranes using Towbine buffer (25 mM Tris, 192 mM glycine, 20% methanol, pH 8.3) at 200 mA for 75 min, in a semi-dry blot system (BioRad). The nitrocellulose membranes were then incubated in the blocking solution (TBS: 50 mM Tris, 150 mM NaCl, pH 7.6, with 0.1% [v/v] Tween 20 and 5% [w/v] nonfat milk), overnight at 4°C, according to Čurin Šerbec *et al*. [34]. Alternatively, a reaction mixture containing the post-nuclear fraction diluted 20-fold in PBS and an appropriate amount of the R30 fraction (0.2 U per 3 µL post-nuclear fraction) was incubated at 92°C for 20 min.

Whole-brain homogenates (2 µL) from TSE infected human, bovine and mouse, and from human Alzheimer’s disease brain, were incubated without or with proteinase K (0.50 µg), for 20 min at 37°C, or without or with the purified pernisine (0.2 U, with the addition of 1 mM CaCl_2_), for 20 min at 92°C. The proteins from the 50-µL reaction mixtures were then dot blotted using standard procedures (BioRad).

The presence of all PrP isoforms and peptides was revealed on the nitrocellulose membranes by immunodetection using the 6H4 anti-PrP monoclonal antibody [35]. The further specific monoclonal antibodies used were: the EB8 anti-PrP monoclonal antibody that recognizes an N-terminal epitope (between residues 20 and 40 of human PrP; our unpublished data); and the C7/5 anti-PrP monoclonal antibody that recognizes a C-terminal epitope (residues 214 to 226 of human PrP [36]). These primary antibodies were revealed using horseradish-peroxidase-conjugated anti-mouse antibodies (Amersham) as the secondary antibodies. The primary antibodies were diluted in TBST (TBS with 0.1% [v/v] Tween 20 and 1% [v/v] nonfat milk) to final concentrations of 0.2 µg/mL (6H4) or 5 µg/mL (EB8 and C7/5). The secondary antibodies were 1500-fold diluted in TBST buffer. Incubations of the membranes with these primary and secondary antibodies were carried out for 1 h at 24°C, with constant agitation. The overnight blocking and the incubation steps were followed by washing steps in TBS with 0.1% (v/v) Tween 20, as two rapid washes, one 15-min wash, and two 5-min washes. After the final washing step, the standard ECL procedure was performed (GE Healthcare), followed by 15 min exposure of the membrane to an ECL film.

### Phosphotungstate Precipitation

The brain-tissue homogenates (post-nuclear fractions; see above) were subjected to proteinase K or R30 fraction digestion before the sodium phosphotungstate (NaPTA) precipitation, with 125 µL digested for 20 min with either proteinase K at 37°C (final concentration, 50 µg/mL) or the R30 fraction at 92°C. For the negative control, aliquots of 25 µL post-nuclear fraction were incubated in the absence of any enzyme activity at 37°C and 92°C. The NaPTA precipitation was performed according to Wadsworth *et al*. [37]. An equal volume of 4% sarcosyl in PBS was added to the enzyme-digested samples and incubated for 15 min at 37°C, with constant agitation. Then, the samples were adjusted to a final concentration of 500 U/mL benzonase (Sigma Aldrich) and 1 mM MgCl_2_ (Sigma Aldrich), and incubated for 30 min at 37°C. Then 27.5 µL PBS and 47.5 µL complete TM-Mini 7× stock (Roche) were added to the enzyme-treated samples, and 5-fold lower amounts to the non-treated samples. Subsequently, 5 µL 4% (w/v) sodium phosphotungstatic acid in 170 mM MgCl_2_ (pH 7.4) was added to the final concentration of 0.3% (w/v) sodium phosphotungstatic acid. The samples were incubated for 30 min at 37°C, with constant agitation, and then centrifuged (14000×*g*, 30 min). The precipitation was also performed for the control samples, with 5-fold lower volumes of reagents used, as compared to the samples treated with the enzymes. The supernatants from the precipitation were discarded, and the pellets were resuspended in 20 µL 0.1% sarcosyl in PBS (pH 7.4). Finally, 10 µL of 3× loading buffer, containing 16% 2-mercaptoethanol, was added.

### Additional Purification of the R30 Proteolytically Active Fraction

The proteolytically active R30 fraction from the Superdex 200 preparative grade gel filtration column was further purified, to identify the active protease(s). The next step of purification/separation of this fraction was using a MonoQ 4.6/100 ion-exchange gel column (GE Healthcare) on a FPLC system. The column was eluted with 50 mM Tris/HCl, pH 8.0, with a linear gradient of NaCl from 0.0 M to 1.0 M at a flow rate of 1 mL/min. The active fraction, which eluted at around 0.5 M NaCl, was ultrafiltered and concentrated with an Ultra-15 Amicon centrifugal filter unit (Millipore) with PBS. The purity of the enzyme activity was analyzed by 12% SDS-PAGE. The proteolytic activity was tested on zymogram gels, as described below.

### Qualitative Proteolytic Activity Assay

To assay the proteolytic activity of specified samples, we followed the zymography procedure described by Foophow *et al*. [38], which is based on zymogram gels (12% SDS-PAGE gels containing 0.1% [w/v] casein (Sigma Aldrich) as substrate). Samples (0.5 µg) were applied to these casein-containing zymogram gels and electrophoresed at a constant 125 V for 70 min. The gels were then soaked in 2.5% (v/v) Triton X-100 on a shaker for 60 min, washed twice with 50 mM Tris/HCl, pH 8.0, containing 1 mM CaCl_2_, and incubated in the same buffer for 4 h at 80°C. The proteolytic activity was visualized as clear bands on the gel against a blue background, using Simplyblue staining.

### Quantitative Proteolytic Activity Assay

The proteolytic activities of specified fractions were determined using the azocasein assay described by Charney *et al*. [39], with some modifications. The protein concentrations were determined by the Bradford method [40], using the BioRad Protein Assay (BioRad) with bovine serum albumin as the standard. Briefly, the reaction mixtures were prepared in PCR tubes and contained 40 µL 50 mM Tris/HCl, pH 8.0, 50 µL 0.2% (w/v) azocasein (Sigma Aldrich) in the same buffer and 10 µL sample (1 µg protein). After 20 min at 92°C, the reactions were stopped by adding 50 µL 15% (w/v) trichloroacetic acid. The samples were kept at 4°C for 10 min, and then centrifuged at 10000×*g* for 10 min. The absorbance of the supernatants was measured at 366 nm against the blank (complete reaction mixture stopped before incubation at 92°C). One unit of protease activity was defined as the amount of enzyme that yielded an increase in A_366_ of 0.1 O.D. under the relevant experimental conditions. The samples were assayed as triplicates and the standard errors calculated. The maximal activity was defined as the highest activity of the purified pernisine in the absence of CaCl_2_.

### Protease Activity

The azocasein assay described above was used to determine the proteolytic activity optimum of the purified pernisine under the different experimental conditions. Initially, the optimum proteolytic activity of the purified pernisine in the presence of different CaCl_2_ concentrations (0 to 8 mM) was investigated. Then, the effects of different NaCl concentrations (0 to 500 mM) on the proteolytic activity of the purified pernisine were examined. To determine the effects of CaCl_2_ on the temperature optimum, the activity of the purified pernisine was measured in the temperature range from 50°C to 150°C in the absence and presence of 1 mM CaCl_2_. A heating block (StarLab, Germany) was used to incubate the reaction mixture for 20 min at 50°C, 70°C, 80°C, 90°C, 99°C, 110°C, 120°C, 130°C and 150°C. To avoid evaporation, the tubes were sealed with parafilm. A similar experiment was performed for the pH range from pH 2 to pH 13. The buffers used were: 50 mM glycine/HCl (pH 2 to 4), 50 mM HEPES (pH 6 to 8) and 50 mM glycine/NaOH (pH 9 to 13). The pH values at the incubation temperature (92°C) were calculated taking into account the dpH/dT coefficients [41]. Similarly, the thermostability of pernisine was determined by the enzymatic activity measurement at different temperatures (70°C and 90°C) in the presence and absence of Ca^2+^ ions at pH 8.0 for prolonged incubation times (0 min, 20 min, 40 min, 120 min) was measured as previously described Catara *et al.* [42].

The effects on the proteolytic activity of inhibitors, reductants, denaturants and detergent were also analyzed. Samples of the purified pernisine in the reaction mixture were preincubated at room temperature for 10 min prior to the azocasein assays. The inhibitors studied were ethylenediaminetetraacetic acid (EDTA; 1, 10 mM), ethylene glycol-bis(b-aminoethyl ether)-*N, N, N, N*-tetraacetic acid (EGTA; 1, 5 mM), iodoacetamide (IAA; 1 mM) and phenylmethylsulfonyl fluoride (PMSF; 1, 10 mM); the reductants were 2-mercaptoethanol (1%, 5%) and dithiothreitol (DTT; 1, 5 mM); the denaturants were urea (1, 4 M) and guanidinium hydrochloride (GdnHCl; 1, 4 M); and the detergent was SDS (0.1%, 3.0%).

### Protein Identification by Tandem Mass Spectrometry

Selected protein bands from the SDS-PAGE were destained and reduced in 25 mM NH_4_HCO_3_ containing 10 mM DTT, for 45 min at 56°C. They were then alkylated in the same buffer containing 55 mM IAA for 30 min at room temperature. The gel pieces were washed with 25 mM NH_4_HCO_3_ and dried. Trypsin solution (12.5 ng/µL trypsin in 25 mM NH_4_HCO_3_) was added, and the samples were incubated overnight at 37 °C. The digested peptides were extracted from the gel with a mixture of 50% acetonitrile and 5% formic acid. The extracts were concentrated to 10 µL using a Concentrator (Eppendorf model 5301) and analyzed by MS/MS.

The MS/MS analysis was performed using a Proxeon EASY-nLC II HPLC unit (Thermo Scientific) coupled to a LTQ Orbitrap Velos ETD mass spectrometer. The peptide samples were loaded onto an Aquasil C18 Picofrit HPLC column (New Objective), and eluted with a 30-min 3% to 50% acetonitrile gradient at a 350 nL/min flow rate. The mass spectra were analyzed by Proteome Discoverer (Thermo Scientific) using a nonredundant NCBI protein database. Positive hits were re-evaluated by Scaffold (Proteome Software). Hits that showed 100% probability were considered as significant.

## Results

### Initial Temperature- and Time-dependent Digestion of PrP^Sc^ by the R30 Fraction

**Figure 1:**
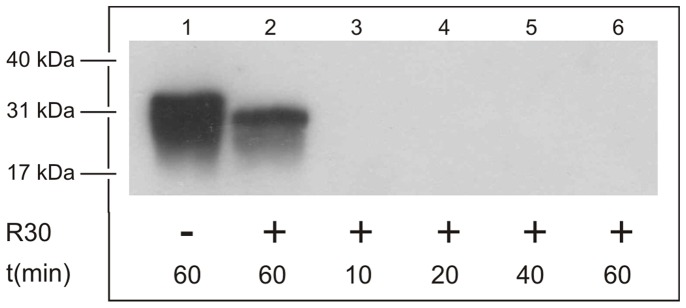
Temperature- and time-dependent degradation of PrP^Sc^ by the R30 fraction, in post-nuclear homogenates from CJD-infected brain. A post-nuclear homogenate from CJD-infected brain was incubated as indicated, without (lane 1) or with (lanes 2–6) the R30 fraction from the Superdex 200 preparative grade gel filtration column, under the following conditions: at room temperature for 60 min (lane 2; RT); or at 92°C for 10 min (lane 3), 20 min (lane 4), 40 min (lane 5), and 60 min (lanes 1, 6). The proteins were separated by SDS-PAGE and transferred to nitrocellulose membrane. The immunoreactive species were detected as described in Materials and Methods, with the 6H4 anti-PrP monoclonal antibody used as the primary antibody.

The effectiveness of the R30 fraction from the Superdex 200 preparative grade gel filtration column for the proteolytic removal of PrP^Sc^ from Creutzfeldt-Jakob disease (CJD)-infected brain homogenates was first estimated. This was followed as the time-dependent degradation of the PrP^Sc^ isoform in the supernatant of the CJD brain homogenates. From Figure 1, it can be seen that the PrP^Sc^ in the supernatant of these brain homogenates is stable over 60 min at 92°C in the absence of the R30 fraction (Fig. 1, lane 1). After addition of the R30 fraction to the reaction mixture, PrP^Sc^ was not detected in the supernatant after just 10 min of incubation at 92°C (Fig. 1, lane 3). On this basis, all of the further incubations were carried out for 20 min at the appropriate temperatures. This proteolysis was due to a hyperthermophilic protease in the R30 fraction, as the degradation of PrP^Sc^ was not complete when the reaction mixture was incubated at room temperature (Fig. 1, lane 2).

### Digestion of PrP^Sc^ by the R30 Fraction

**Figure 2:**
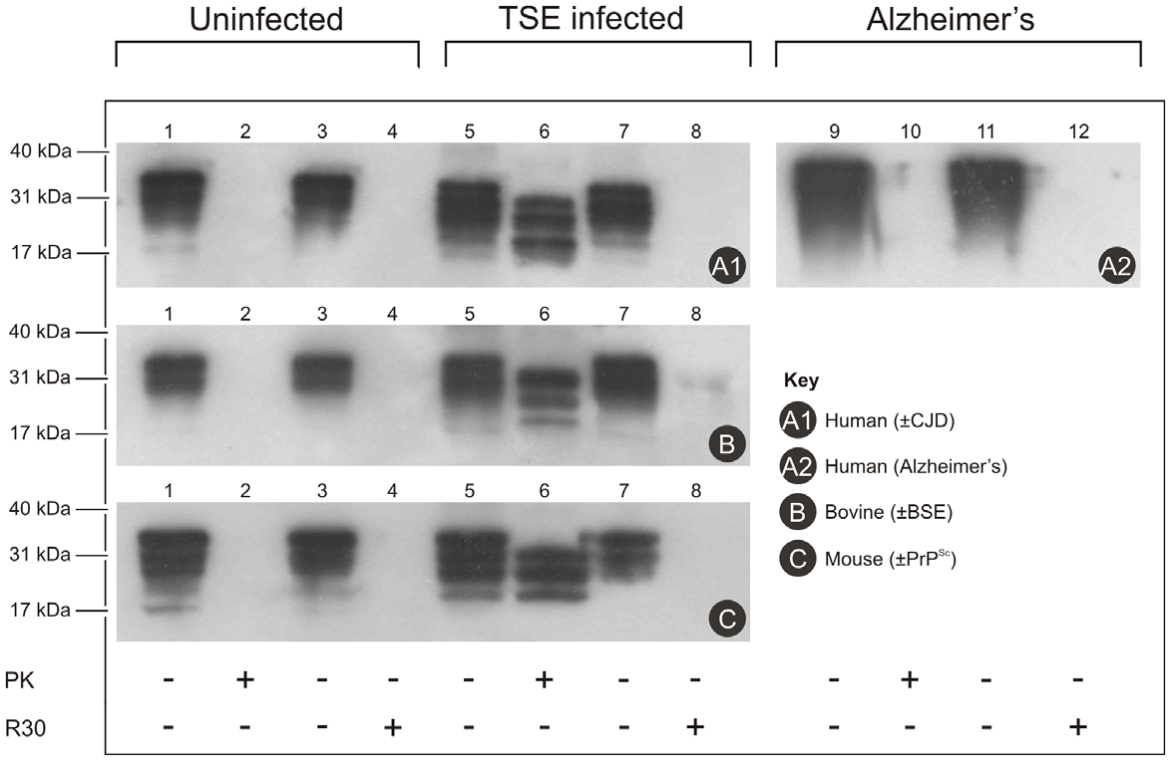
Digestion of PrP^C^ and PrP^Sc^ of different species by the R30 fraction. Post-nuclear homogenates from human (A), bovine (B) and mouse (C) uninfected (lanes 1–4), TSE infected (lanes 5 to 8) and Alzheimer’s disease (lanes 9 to 12) brains were incubated as indicated, without (lanes 1, 5, 9) or with (lanes 2, 6, 10) 0.75 µg proteinase K (PK), for 20 min at 37°C, or without (lanes 3, 7, 11) or with (lanes 4, 8, 12) the R30 fraction from the Superdex 200 preparative grade gel filtration column, for 20 min at 92°C. The proteins were separated by SDS-PAGE and transferred to nitrocellulose membranes. The immunoreactive species were detected as described in Materials and Methods, with the 6H4 anti-PrP monoclonal antibody used as the primary antibody.

Digestion of the cellular prion protein, PrP^C^, by the R30 fraction at 92°C was comparable with that of proteinase K at 37°C (Fig. 2). Proteinase K and the R30 fraction both removed the immunoreactive material from the reaction mixtures to below the detection level of Western blotting (Fig. 2, lanes 2, 4, respectively). Furthermore, a significant difference in proteolytic degradation was obtained when supernatants of CJD brain homogenates were used (Fig. 2, panel A1). After 20 min of incubation of the PrP^Sc^ samples with proteinase K, there was little digestion of the immunoreactive material (Fig. 2, lanes 5, 6). On the contrary, with the R30 fraction, the immunoreactive material from the reaction mixtures was again below the detection level of Western blotting (Fig. 2, lanes 7, 8). The proteolytic activity of the R30 fraction also did not depend on the species of origin of the PrP used (Fig. 2, panels A1, B, C). The activities of proteinase K and the R30 fraction were also tested against the protein from amyloid plaques typical for human Alzheimer’s disease (Fig. 2, panel A2). Here, again, the R30 fraction completely digested the immunoreactive material from the reaction mixtures to below the detection level of Western blotting (Fig. 2, panel A2, lanes 11, 12), although here the same was also seen for proteinase K (Fig. 2, panel A2, lanes 9, 10).

### Detection of Smaller sized Peptides Following PrP^Sc^ Digestion

**Figure 3:**
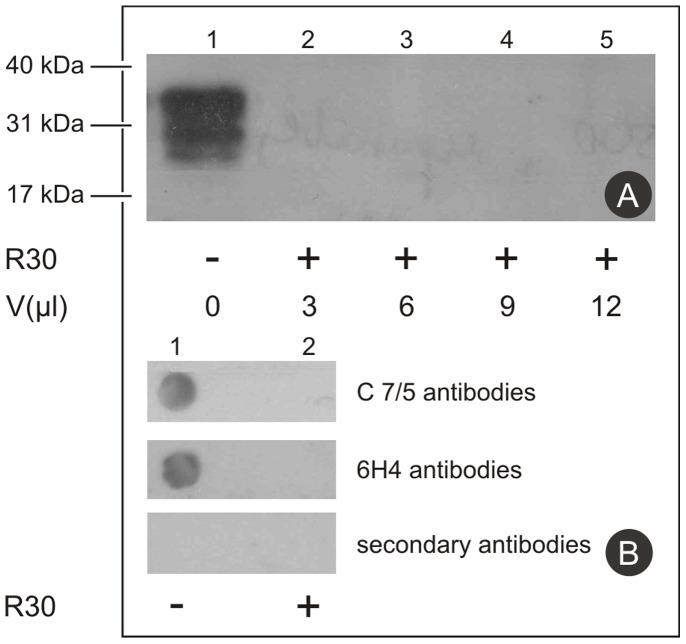
Detection of smaller sized peptides as a result of PrP^Sc^ digestion. **A.** Post-nuclear homogenates from uninfected (lane 1) and CJD-infected (3, 6, 9 and 12 µL; lanes 2–5, respectively) brains were incubated as indicated, without (lane 1) and with the R30 fraction from the Superdex 200 preparative grade gel filtration column, for 20 min at 92°C. The proteins were separated by SDS-PAGE and transferred to nitrocellulose membranes. The immunoreactive species were detected as described in Materials and Methods, with the EB8 anti-PrP (N-terminal) monoclonal antibody used as the primary antibody. **B.** Diluted (20x) post-nuclear homogenates from CJD-infected brain were incubated as indicated, without (lane 1) or with (lane 2) the R30 fraction from the Superdex 200 preparative grade gel filtration column, for 20 min at 92°C. The proteins were dot blotted onto nitrocellulose membranes. The immunoreactive species were detected as described in Materials and Methods, with the C7/5 anti-PrP (C-terminal) and the 6H4 anti-PrP monoclonal antibody used as the primary antibodies, as indicated.

The degradation of PrP^Sc^ by the R30 fraction was further examined with the use of two monoclonal antibodies against different epitopes of PrP^Sc^, for the N-terminal (EB8) and the C-terminal (C7/5). For the detection of smaller peptides that would result from incomplete digestion of PrP^Sc^ at the N-terminal with the EB8 antibodies, no immunoreactive material (and hence no smaller N-terminal fragments) were detected with Western blotting (Fig. 3A, lanes 2–5). Furthermore, even in reaction mixtures with up to 4-fold the original amount of PrP^Sc^-infected brain homogenate supernatant there were still no smaller degradation products of PrP^Sc^ detected following this treatment with the R30 fraction (Fig. 3A, lane 5).

Similarly, for the detection of smaller peptides that would result from incomplete digestion of PrP^Sc^ at its C-terminal, the C7/5 monoclonal antibody was used. Here, dot blot analysis was used, as the C7/5 monoclonal antibody does not bind to the denatured epitope. The PrP^Sc^ isoform was confirmed only in the control reaction mixture, while the addition of the R30 fraction also resulting in the complete disappearance of the signal in the dot blot analysis (Fig. 3B). The appropriate controls with the 6H4 monoclonal antibody and the secondary antibodies alone were incorporated in the same experiment, and gave the expected results (Fig. 3B). The 6H4 monoclonal antibody reacted only with the undigested PrP^Sc^ samples, and the secondary antibodies did not react with either of these samples.

**Figure 4:**
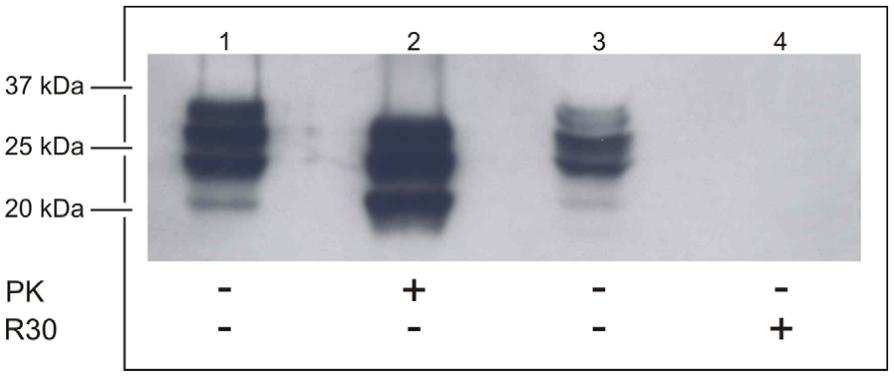
Detection of phosphotungstate-precipitable PrP. Post-nuclear homogenates from CJD-infected brain were incubated as indicated, without (lane 1) or with (lane 2) 0.75 µg proteinase K (PK), for 20 min at 37°C, or without (lane 3) or with (lane 4) the R30 fraction from the Superdex 200 preparative grade gel filtration column, for 20 min at 92°C. Following NaPTA precipitation as described in Materials and Methods, the proteins were separated by SDS-PAGE and transferred to nitrocellulose membranes. The immunoreactive species were detected as described in Materials and Methods, with the 6H4 anti-PrP monoclonal antibody.

The extent of degradation of PrP^Sc^ by the R30 fraction was further estimated using NaPTA precipitation. The post-nuclear fractions from CJD-infected brain homogenates were incubated for 20 min in the absence and presence (5-fold greater volume) of proteinase K (at 37°C) or the R30 fraction (at 92°C), and then subjected to the NaPTA precipitation procedure (Fig. 4, lanes 1, 2, and lanes 3, 4, respectively). The proteins were separated by SDS-PAGE and then transferred to nitrocellulose membranes. The immunoreactive species were detected using the 6H4 monoclonal antibody. From the intensities of the bands in Figure 4 for lanes 1 and 3, although there was less human PrP^Sc^ in the sample than in that incubated with the R30 fraction at 92°C (Fig. 4, lane 3), as compared to proteinase K at 37°C (Fig. 4, lane 1), there were no detectable fragments from the proteolytic degradation of PrP^Sc^ by the R30 fraction in this Western blotting (Fig. 4, lane 4).

### Identification of Proteolytically Active Purified Pernisine from the R30 Fraction

**Figure 5:**
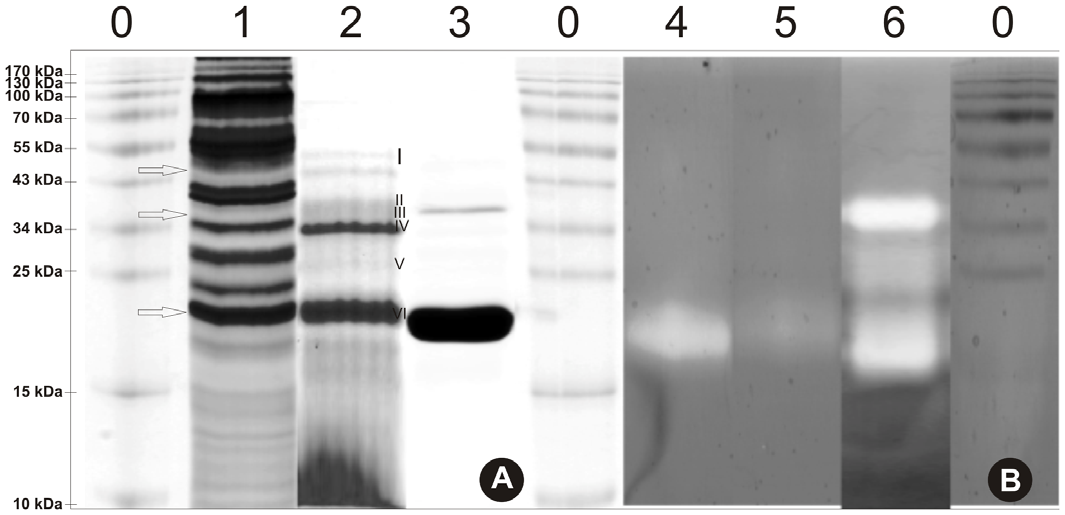
SDS-PAGE analysis and zymography through the purification steps of the medium from *A. pernix*. Representative gels of medium samples through the purification, following electrophoresis on standard 12% SDS-PAGE (A) and on 12% SDS-PAGE with casein as substrate for zymography activity (B: 4 h at 80°C), with staining with Simplyblue dye. Lanes 0, protein MW markers (as indicated left); lanes 1 and 4, concentrated medium fraction; lanes 2 and 5, R30 fraction from the Superdex 200 preparative grade gel filtration column; and lanes 3 and 6, the post-monoQ purified pernisine fraction (see Table 1). Lane 2, I-VI, selected protein bands of R30 fraction taken for MS/MS analysis.

The R30 fraction from the Superdex 200 preparative grade gel filtration column was further purified using a monoQ column, as described in Materials and Methods. The purification steps from the crude extract to this purified pernisine are summarized in Table 1. This pernisine protease was purified 9.7-fold, with a final 17% yield, and with a specific activity of 2091 U/mg protein. In Fraction R30 there are clearly visible two distinctive protein bands with molecular masses (MW) of 34 kDa and 23 kDa (Fig. 5A, lane 2, IV, VI) and low intensity bands at 46 kDa (lane 2, I) and 10 kDa (Fig. 5A, lane 2). Further purification of this R30 fraction using monoQ ion-exchange chromatography highlighted protein bands at 23 kDa and at around 34 kDa (Fig. 5A, lane 3, III, IV). Activity staining showed that the R30 fraction and the additionally purified post-monoQ fraction both contained a proteolytically active band, as estimated from a zymogram gel (Fig. 5B, lanes 5, 6), with a MW of around 23 kDa (Fig. 5A) and at 34 kDa, observed only in purified pernisine.

**Table 1:** Summary of the steps for the purification and identification of the pernisine from the growth medium of *A. pernix* K1.

Purification step	Specific activity[U/mg protein]#	Total protein [mg]	Total activity [U]#	Yield [%]	Purification [fold]
Concentrated crude extract*	216	25	5400	100	1
Post S-200 (R30 fraction)§	727	6.15	4470	83	3.4
Post monoQ (purified pernisine)§	2091	0.44	920	17	9.7

* Note: during concentration using 10-kDa cut-off membranes, low molecular mass proteins were lost.

# One unit of protease activity is defined as the amount of enzyme that yields an increase in A_366_ of 0.1 O.D. under the relevant experimental conditions.

§ As used in the present study.

Mass spectrometry analysis revealed that bands I and IV of Figure 5A, lane 2, contained fragments from pernisine. The other bands contained various fragments from other *A. pernix* proteins: ABC-transporter like proteins, hypothetical proteins APE_0061 and APE_1117, and a surface-layer protein (Table S1). All of these identified proteins are part of the extracellular fraction of proteins from *A. pernix* [43], [44].

Data from the SDS-PAGE, MS/MS analysis and zymography imply that this purified pernisine has a proregion that is autocleaved during maturation. Pernisine has a MW of 44 kDa, as calculated from its amino-acid sequence. Based on MS/MS data (Table S1), the protein bands with MW of 46 kDa and 34 kDa in R30 fraction (Fig. 5A, lane 2) as well as the protein bands at 34 kDa and 23 kDa in the purified pernisine (Fig. 5A, lane 3) correspond to the same enzyme - pernisine. According to Figure 5, the purified pernisine is in active mature form at 23 kDa and in pre-form at 34 kDa (Fig. 5A, lane 6). Thus, it appears that the purified pernisin is a mixture of two different active forms, which will have to be further analysed.

### Digestion of PrP^Sc^ by the Purified Pernisine

**Figure 6:**
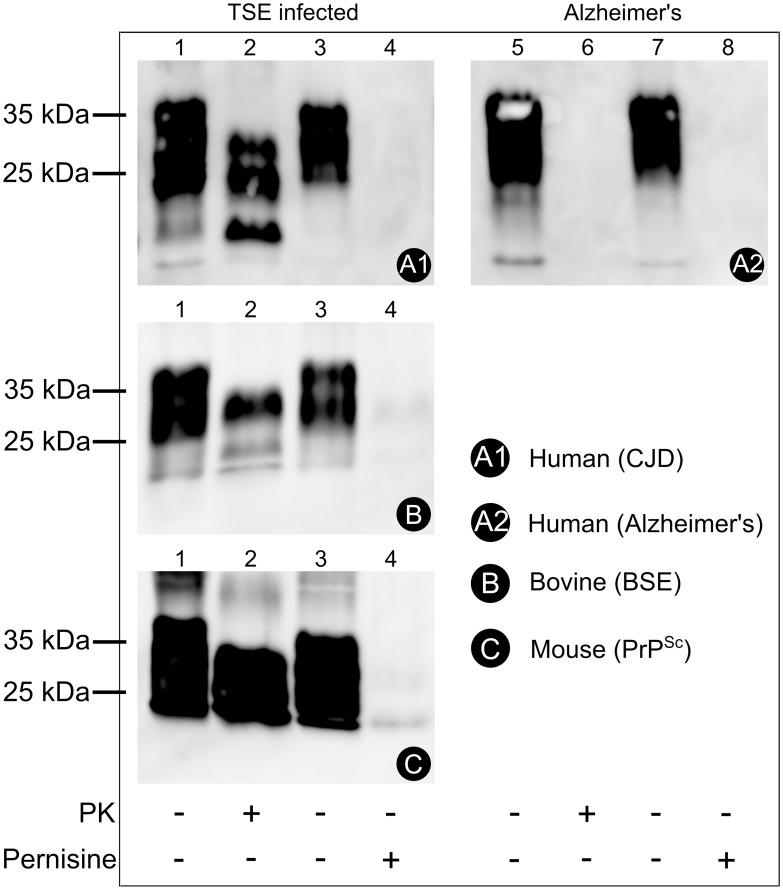
Digestion of PrP^C^ and PrP^Sc^ of different species by purified pernisine. Post-nuclear homogenates from human (A), bovine (B) and mouse (C), TSE infected (lanes 1 to 4) and Alzheimer’s disease (lanes 5 to 8) brains were incubated as indicated, without (lanes 1, 5) or with (lanes 2, 6) 0.75 µg proteinase K (PK), for 20 min at 37°C, or without (lanes 3, 7) or with (lanes 4, 8) pernisine for 20 min at 92°C. The proteins were separated by SDS-PAGE and transferred to nitrocellulose membranes. The immunoreactive species were detected as described in Materials and Methods, with the 6H4 anti-PrP monoclonal antibody used as the primary antibody.

As described for fraction R30, digestion of the cellular prion protein, PrP^C^, by the purified pernisine at 92°C was comparable with that of proteinase K at 37°C (Fig. 6). A significant difference in the proteolytic degradation was obtained when supernatants (data not shown) of CJD brain or complete homogenates were used (Fig. 6, panel A1). After 20 min of incubation of the PrP^Sc^ samples with proteinase K, there was little digestion of the immunoreactive material (Fig. 6, lanes 2). On the contrary, with the pernisine, the immunoreactive material from the reaction mixtures was again below the detection level of Western blotting (Fig. 6, lanes 4). The proteolytic activity of pernisine also did not depend on the species of origin of the PrP used (Fig. 6, panels A1, B, C). The activities of proteinase K and pernisine were also tested against the protein from amyloid plaques typical for human Alzheimer’s disease (Fig. 6, panel A2). Here, again, the pernisine digested the immunoreactive material from the reaction mixtures to below the detection level of Western blotting (Fig. 3, panel A2, lane 8), although the same was observed for proteinase K (Fig. 3, panel A2, lane 6).

### Effects of CaCl_2_ and NaCl on the Enzymatic Activity

**Figure 7:**
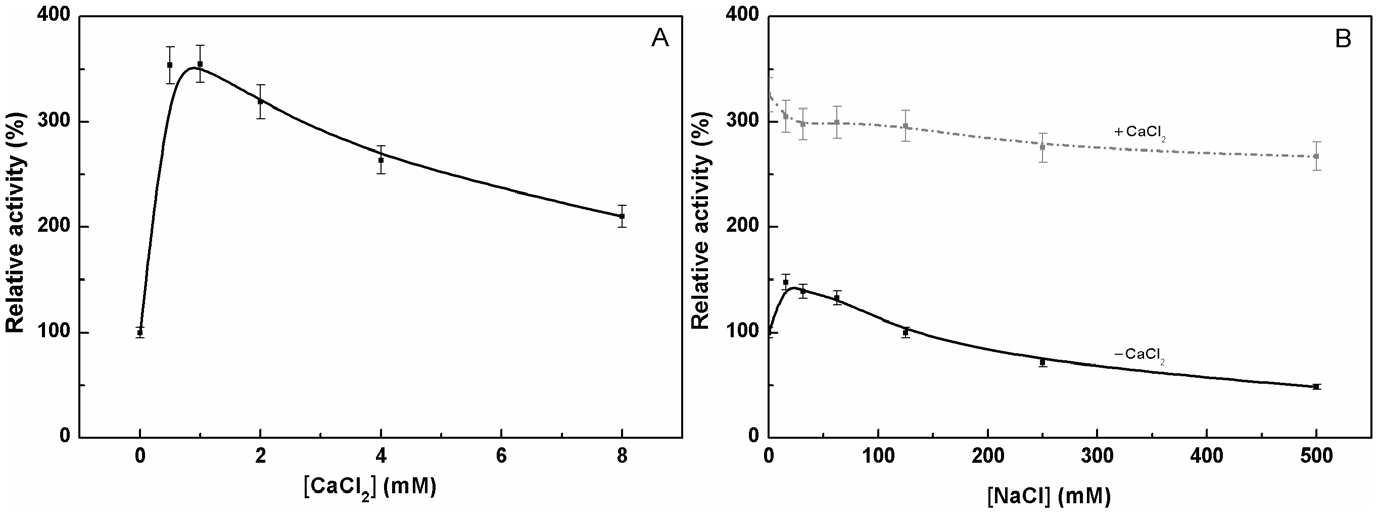
Azocasein assays of the purified pernisine: effects of CaCl_2_ and NaCl. Relative activities of the purified pernisine according to increasing CaCl_2_ concentrations (A), and according to increasing NaCl concentrations in the presence (gray, dot-dash line) and absence (black line) of 1 mM CaCl_2_ (B). Assays were carried out in 50 mM Tris/HCl, pH 8.0, with 0.1% (w/v) azocasein, for 20 min at 92°C. Data are means ± SD from three independent experiments. The lines shown represent the best fit of the data according to OriginPro 8.0 program.

Figure 7A shows the results for the relative activity of this purified pernisine at 92°C and pH 8.0 according to the addition of increasing concentrations of CaCl_2_ (0–8 mM). The maximum enhancement of the purified pernisine activity was observed at around 1 mM CaCl_2_. Further increasing the CaCl_2_ above 1 mM led to a gradual decline in this enhanced activity. Similar enhancement of CaCl_2_ on enzyme activity was reported for aeropyrolysine, another heat-stable extracellular protease from *A. pernix* K1 [45].

The effects of increasing concentrations of NaCl (0–500 mM) on the relative activity of the purified pernisine in the absence and presence of 1 mM CaCl_2_ are presented in Figure 6B. The maximal activity in the absence of added CaCl_2_ was seen with 20 mM NaCl. As the concentration of NaCl was increased from 0 mM to 500 mM, the proteolytic activity of the purified pernisine decreased by more than 50%. The initially higher activity in the presence of 1 mM CaCl_2_ showed a decrease with the addition of NaCl, although this was seen as only around 10% over the same concentration range of NaCl (Fig. 7B). The maximal activity of the purified pernisine seen for 20 mM NaCl in the absence of 1 mM CaCl_2_ increased to more than two-fold after the addition of CaCl_2_, with even larger relative enhancement by CaCl_2_ at higher NaCl concentrations (Fig. 7B).

Single charged electrolytes like NaCl can have influences on charged groups on the surface of a protein [46], which can be especially important if these are charged groups near to a catalytically active site. The further increases in the concentration of NaCl (as ionic strength) might affect such charges near to the active site. Indeed, the net charge of a protein depends on the ionic strength, pH, salt type, salt concentration, and ionic valence. Altogether, changes to the charge of a protein can have influences on its thermostability, solubility and biological activity [46]. We can also conclude here that NaCl and CaCl_2_ together do not show any cumulative effects, as they appear to separately affect the activity, reflecting their relatively different effects on enzyme stabilization and/or kinetics.

### Effects of Temperature and pH on the Enzymatic Activity

**Figure 8:**
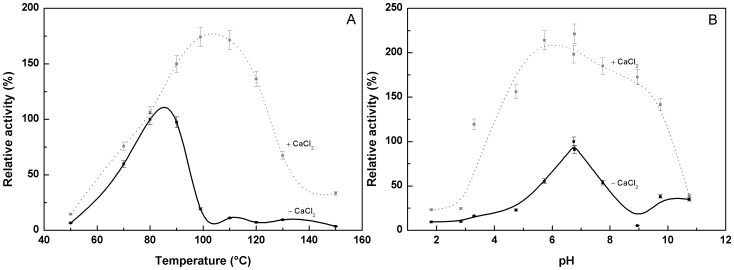
Azocasein assays of the purified pernisine: effects of temperature and pH. Relative activities of the purified pernisine in the presence (gray dashed lines) and absence (black lines) of 1 mM CaCl_2_ according to temperature (**A**), in 50 mM Tris/HCl, pH 8.0, and according to pH (**B**), in 50 mM glycine/HCl, pH 2 to 5, 50 mM HEPES, pH 6 to 8, or 50 mM glycine/NaOH, pH 9 to 11, at 92°C. Assays were carried out for 20 min. Data are means ± SD from three independent experiments. The lines shown represent the best fit of the data according to OriginPro 8.0 program.

The effect of temperature on the proteolytic activity of the purified pernisine was examined in the absence and presence of 1 mM CaCl_2_ (Fig. 8A), using the azocasein assay at pH 8.0 (in 50 mM Tris/HCl). The purified pernisine showed proteolytic activity in the broad temperature range from 70°C to 95°C, with the maximal activity at around 85.0±0.5°C. In the presence of 1 mM CaCl_2_, the proteolytic activity of pernisine was enhanced, to reach around 75% greater activity, with the maximum activity at 105.0±0.5°C (Fig. 8A).

The shift in the temperature optimum in the presence of CaCl_2_ has already been described for pernisine [42] and for some other proteins: e.g. aeropyrolysine [45], recombinant tengconlysine [47] and aqualysin I [48]. The effects of pH on the enzymatic activity of the purified pernisine at 92°C are shown in Figure 8B. The activity of pernisine at 92°C was detected (>20%) in the broad range from pH 3.5 to 8.0, with a pH optimum at 7.0±0.2. In the presence of 1 mM CaCl_2_, there was significantly enhanced activity of the purified pernisine observed in the wide pH range from 3 to 10 (Fig. 8B), with the pH optimum at 6.5.

**Figure 9:**
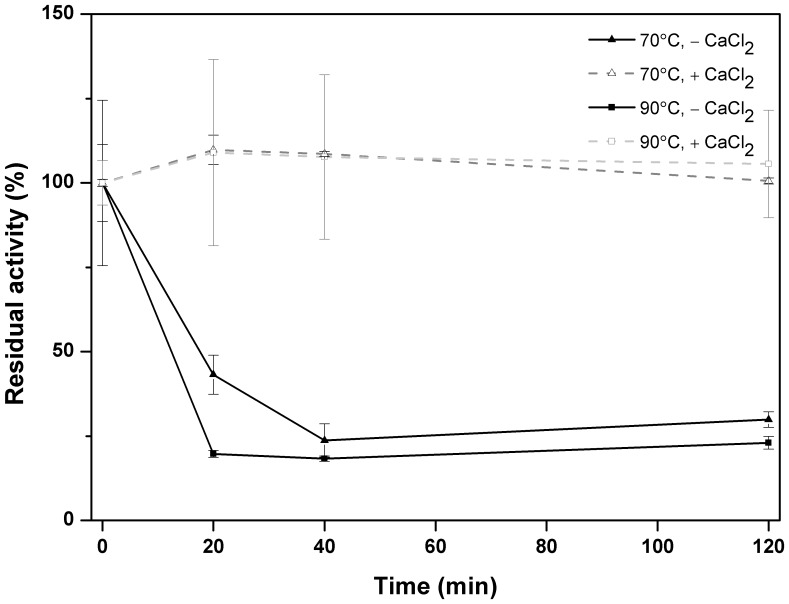
Thermostability of pernisine. Residual activity of pernisine incubated at different temperatures (70°C and 90°C) in the presence and absence of Ca^2+^ ions at pH 8.0 for prolonged incubation times (0 min, 20 min, 40 min, 120 min) as marked.

The enzymatic activity of pernisine at different temperatures (70°C and 90°C) in the presence and absence of Ca^2+^ ions at pH 8.0 for prolonged incubation times (0 min, 20 min, 40 min, 120 min) showed that pernisine remains proteolytic activity in the presence of Ca^2+^ ions. In the absence of Ca^2+^ decreasing of residual proteolytic activity over time was observed (Figure 9). Based on these observations we can conclude that pernisine is thermally stable protease.

### Effects of Protease Inhibitors on the Enzymatic Activity

Different inhibitors (Table 2) were used to test for their effects on the protease activity of the purified pernisine. These included inhibitors of metalloproteases and serine and cysteine proteases, as specified in Table 2. EDTA, EGTA and PMSF had the greatest inhibitory effects on the purified pernisine activity, which confirms that it is a metalloprotease and serine protease. Addition of 1 mM EDTA or EGTA to the purified pernisine in the absence of added CaCl_2_ effectively completely inhibited the protease activity of the purified pernisine (Table 2). However, in the presence of 1 mM CaCl_2_, 1 mM EDTA and EGTA had no significant effects, with the need for higher (5 mM) EDTA and EGTA for complete inhibition of the purified pernisine. In contrast, as a serine protease inhibitor, PMSF (1 mM, 10 mM) effectively blocked this activity both in the presence and absence of 1 mM CaCl_2_, while IAA as a cysteine protease inhibitor showed no inhibition here, even at 10 mM.

**Table 2:** Residual protease activity of the purified pernisine in the absence and presence of CaCl_2_ and protease inhibitors.

Inhibitor	Concentrationadded [mM]	Class of inhibitor	Residual protease activity without CaCl_2_ [%]	Residual protease activity with 1 mM CaCl_2_ [%]
None	–	–	100	216
EDTA	1	Metalloprotease	3	202
	5		0	1
EGTA	1	Metalloprotease	1	197
	5		0	3
PMSF	1	Serine protease	7	15
	10		2	6
Iodoacetamide	10	Cysteine protease	103	198

Its is likely that EDTA and EGTA affect this purified pernisine activity not only by extracting the Ca^2+^ ions, which could lead to denaturation of the protein, but also by influencing the autolysis of the purified pernisine, as implied by Catara *et al*. [42]. Thus, it is likely that some Ca^2+^ ions will already be coordinated with this purified pernisine, similar to what has been shown for Tk-SP: Ca^2+^ is bound too tightly to Tk-SP to be removed with extensive dialysis against Ca^2+^-free buffer, but it can be removed by treatment with 10 mM EDTA [49].

### Effects of Denaturing Agents on the Enzymatic Activity

Table 3 gives the data for the residual purified pernisine activity in the presence of further reagents: two reductants (DTT and 2-mercaptoethanol), two denaturants (GdnHCl and urea) and a detergent (SDS). With the purified pernisine in the absence of 1 mM CaCl_2_, at both 1 mM and 5 mM, DTT showed about 65% inhibition of the purified pernisine activity, which was slightly reduced, to around 45% inhibition, in the presence of 1 mM CaCl_2_ (Table 3). Similar effects were seen for 2-mercaptoethanol, with about 55% and 35% inhibition, respectively (Table 3).

**Table 3:** Residual protease activity of the purified pernisine in the absence and presence of CaCl_2_ and reductants, denaturants and detergent.

Reagent	Concentration added	Class of reagent	Residual protease activity without CaCl_2_ [%]	Residual protease activity with 1 mM CaCl_2_ [%]
None	–	–	100	201
Dithiothreitol	1 mM	Reductant	35	56
	5 mM		34	52
2-mercaptoethanol	1%	Reductant	48	70
	5%		39	62
Guanidine HCl	1 M	Denaturant	27	133
	4 M		35	306
Urea	1 M	Denaturant	52	104
	4 M		50	94
SDS	0.1%	Detergent	69	132
	3.0%		20	68

For the denaturants, in the presence of 1 mM CaCl_2_, 1 M GdnHCl showed greater inhibition of the purified pernisine compared to 4 M urea (residual activities, 27% *vs*. 50%, respectively). Of note, at the higher GdnHCl of 4 M, this also decreases the pH, which reflects on the change in the enzyme activity. Similarly, this might also be related to the case of addition of CaCl_2_ here, where 4 M GdnHCl increased the enzyme activity over and above the Ca^2+^ effect (Table 3). However, it is also known that some denaturants can affect enzyme active sites, promoting faster catalysis [47].

The purified pernisine remains relatively active with a low SDS concentration (0.1%), both in the absence and presence of 1 mM CaCl_2_, with residual activity also remaining with the addition of 3% SDS (Table 3).

## Discussion

In the present study, we have shown that pernisine is the proteolytic enzyme that we have purified from the growth medium of *A. pernix* and that can digest PrP^Sc^ that is resistant to degradation by proteinase K. The R30 fraction and the purified pernisine can digest PrP^Sc^ from different species (bovine, mouse, human) (Figures 2, 6 A1), as well as protein plaques in Alzheimer disease (Figures 2, 6, A2). Thus our analysis of the fraction (R30) and purified enzyme (Table S1) by MS/MS identified pernisine as the active enzyme.

**Figure 10:**
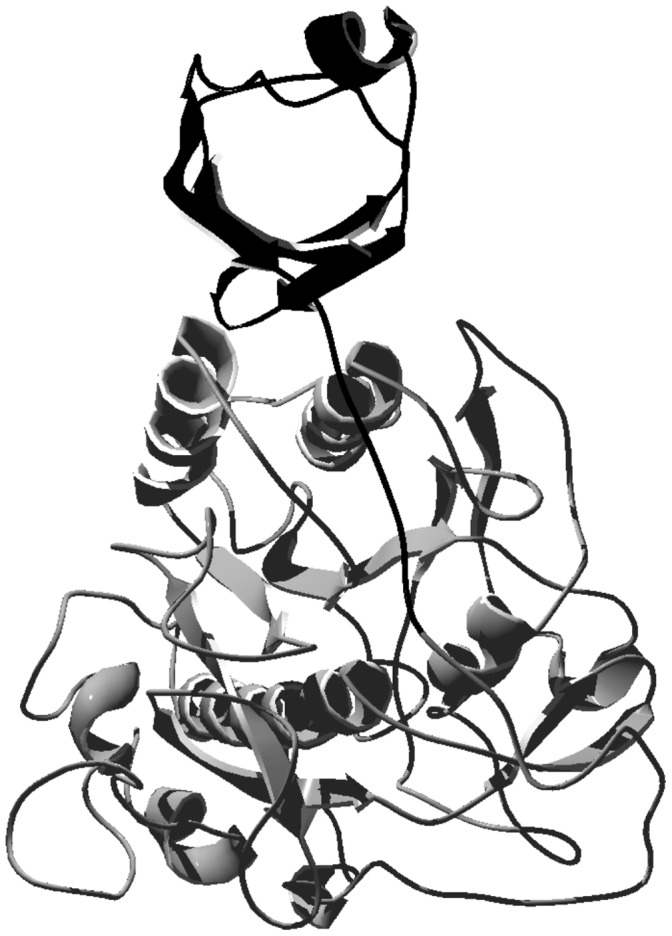
Model of the three-dimensional structure of pernisine. Proposed proregion (black; Val1-Met62) and mature (gray; Ala63-Val396) forms. Model structure was built using the Geno3D2 program. Note: part of the N-terminal of the amino-acid sequence (M1-V32) is missing.

Catara *et al*. [42] identified pernisine by SDS-PAGE and gel filtration, and they determined its proteolytic activity by SDS-PAGE with casein overlay. In their study, the recombinant pernisine was expressed in *Escherichia coli*, and the data suggested that it has a proregion. Our data in the present study also indicate that the purified pernisine is likely to have a proregion, as was seen for the similar protease Tk-subtilisin [50], and for other bacterial subtilisins [38]. The alignment of the amino-sequence of pernisine with Tk-subtilisin showed 51.7% identity and 66.5% consensus in their sequences. Tk-subtilisin has a signal sequence M^1^-A^24^ and a prosequence G^25^-L^93^. Analysis of the amino-sequence of pernisine using the SignaIP 3.0 program, which uses neural networks and hidden Markov models that are trained on eukaryotes, leads us to propose that pernisine has a M^1^-G^24^ signal sequence. From an alignment of pernisine with Tk-subtilisin, we propose a prosequence of S^25^-M^94^. This prediction is in agreement with the data from SDS-PAGE of the purified pernisine, with the two bands seen corresponding the MWs of 45 kDa with the proregion, and 34 kDa without it (Figure 10). The active form of pernisine observed at 23 kDa at higher protein concentrations could be the result of further autocatalytic activity of pernisine, similarly as it was reported before for Tk-SP [38]. By following the changes in the enzymatic activity under a broad range of temperature for prolonged incubation times (Figure 9), we can conclude that pernisine is a thermostable metalloprotease that is compatible with other thermostable enzymes that have already been described, such as: Tk-subtilisin and Tk-SP from *T. kodakaraensis* [49], aerolysine from *A. pernix* [45], and Aqualysin I from *Thermus aquaticus* YT-1 [48].

Pernisine is a serine metalloprotease that is active in the temperature range from 60°C to 99°C, in the pH range from 3.5 to 8.0, and at NaCl concentrations from 0 mM to 500 mM. Its enzymatic activity optimum is at 85.0±0.5°C, pH 7.0±0.25 and 20 mM NaCl. The presence of 1 mM CaCl_2_ increases the enzymatic activity to azocasein by two-fold, and shifts the activity optimum to 105±1°C and the pH optimum to 6.5±0.2. In the presence of 1 mM CaCl_2_, the purified pernisine maintains its enzymatic activity at NaCl concentrations of 0 mM to 500 mM, while with 5 mM EDTA or EGTA, the purified pernisine is completely inhibited. This purified pernisine activity is not restored here even after addition of 1 mM CaCl_2_. Furthermore, the purified pernisine is inhibited by the serine protease inhibitor PMSF.

Based on our biochemical characterization of the purified pernisine, we can conclude that CaCl_2_ has a crucial role in the proteolytic activity at higher temperatures. This broad range of pH and temperature activity of pernisine can thus be exploited for different biotechnological and industrial applications in the food industry, in decontamination processes of wastes, in the leather industry, and in the degradation of infective protein aggregates [38].

## Supporting Information

Table S1 **Proteins identification by tandem mass spectrometry in fraction R30 and post monoQ fraction-purified pernisine.** Bands marked I-VI and monoQ fraction of purified pernisine in Figure 5A were analyzed by MS/MS and data of identified proteins of specific bands are presented in the table.
(XLS)
